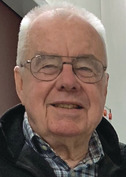# A Case for Bond‐Network Analysis in the Synthesis of Bridged Polycyclic Complex Molecules: Hetidine and Hetisine Diterpenoid Alkaloids

**DOI:** 10.1002/anie.201909656

**Published:** 2020-04-15

**Authors:** Nicolle A. Doering, Richmond Sarpong, Reinhard W. Hoffmann

**Affiliations:** ^1^ Latimer Hall Department of Chemistry University of California, Berkeley Berkeley CA 94720 USA; ^2^ Fachbereich Chemie der Philipps Universität Marburg 35032 Marburg Germany

**Keywords:** alkaloids, polycycles, synthesis design, terpenoids, total synthesis

## Abstract

A key challenge in the synthesis of diterpenoid alkaloids lies in identifying strategies that rapidly construct their multiply bridged polycyclic skeletons. Existing approaches to these structurally intricate secondary metabolites are discussed in the context of a “bond‐network analysis” of molecular frameworks, which was originally devised by Corey some 40 years ago. The retrosynthesis plans that emerge from a topological analysis of the highly bridged frameworks of the diterpenoid alkaloids are discussed in the context of eight recent syntheses of hetidine and hetisine natural products and their derivatives. This Minireview highlights the extent to which network analyses of the type described here sufficed for designing synthesis plans, as well as areas where they had to be amalgamated with functional group oriented synthetic planning considerations.

## Graphical abstract

**A key challenge** in the synthesis of diterpenoid alkaloids lies in identifying efficient strategies to construct their multiply bridged polycyclic skeletons. Approaches from eight recent syntheses are discussed in the context of “bond‐network analyses”, highlighting the efficacy of bond‐network analyses for synthesis planning, as well as where other considerations had to be incorporated.

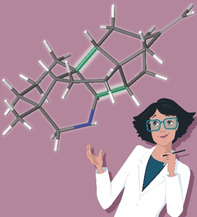

## 1 Introduction

### 1.1 Retrosynthesis and Bondsets

Retrosynthesis has been adopted as the standard approach to identifying synthetic strategies to construct organic molecules. For many practitioners of organic synthesis, generating a synthetic plan for the preparation of a complex molecule without undertaking a retrosynthetic analysis is unfathomable.^1^ Retrosynthesis, which involves breaking the bonds that will be formed in the forward (synthetic) direction, is often guided by knowledge of known transformations to forge those bonds. Over the years, the way in which retrosynthetic analyses are conducted has been formalized, led principally by the “Logic of Chemical Synthesis” introduced by Corey.^2^

In one approach to retrosynthesis, the bonds chosen for disconnection are marked on the structure of the target molecule and designated as the bondset for the intended synthesis.^3^ The selection of the individual bonds to be included in a bondset hinges upon the topology of the target framework, as well as upon consideration of which functional groups in the target structure may be leveraged to achieve bond formation in the course of the forward synthesis. Thus, consideration of both how to minimize structural complexity in the retrosynthetic direction, as well as what reactions can be employed in the synthetic direction, are taken into account in retrosynthetic analyses.^2^

Since a bondset does not specify the sequence in which individual bonds are to be forged in the synthetic direction, the order in which the bonds are broken must also be considered. In the end, the sequence that emerges for bond formation should correspond to an efficient forward synthesis, in which the attributes of a convergent versus linear synthesis are maximized.^3^ In general, step count in complex molecule synthesis is minimized when an exponential increase in the structural complexity of the intermediates is achieved en route to the target.^4^ In the context of a retrosynthesis, this requires judicious choice of disconnections that will lead to maximal simplification at each stage.

Ultimately, the step count and elegance of a synthesis are heavily influenced by the choice of which bonds are placed in the bondset and when they are generated in the forward synthesis.

### 1.2 Diterpenoid Alkaloids: A Class of Highly Bridged Natural Products

**Figure 1:**
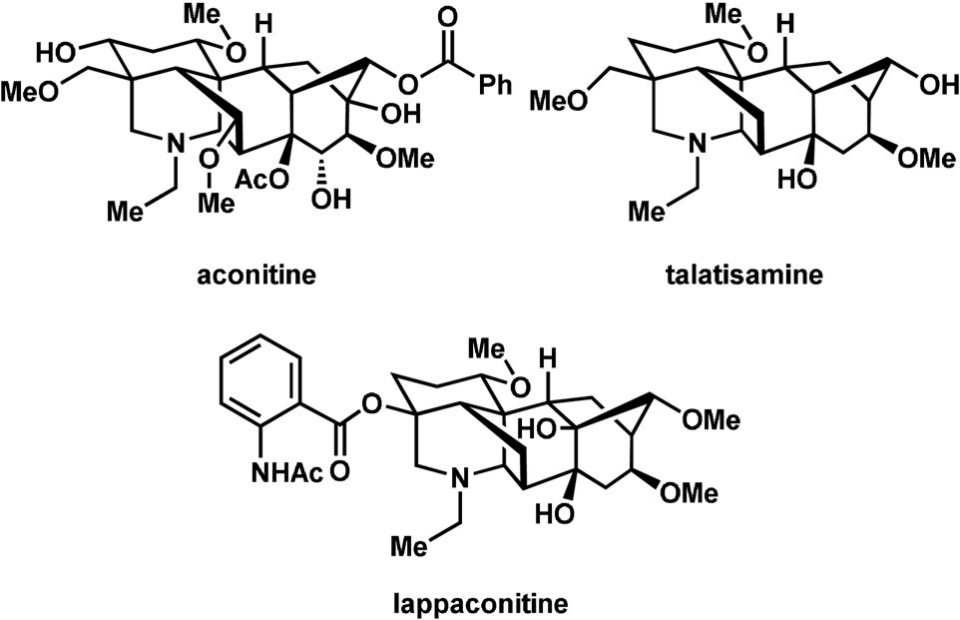
Aconitine‐type diterpenoid alkaloids with known biological activity.

The diterpenoid alkaloids are a topologically complex group of molecules that have been shown to possess interesting biological activity, primarily in modulating voltage‐gated ion channels.^5^ The most well‐known of the diterpenoid alkaloids, the toxin aconitine, has long been recognized as an activator of sodium ion channels,^6^ whereas the related alkaloid lappaconitine is an anti‐arrhythmic agent that functions by blocking sodium ion channels (Figure 1).^7^ On the other hand, talatisamine instead blocks potassium ion channels. This contrasting biological activity has raised questions about structure–activity relationships among diterpenoid alkaloids and their derivatives. This myriad activity, in conjunction with their impressive architecture, has sparked interest in the total synthesis of diterpenoid alkaloids.

**Figure 2:**
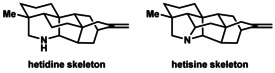
Hetidine and hetisine skeletons.

Although several hundred diterpenoid alkaloids have been structurally characterized,^5^ only a handful of these intricate molecules had been synthesized in the laboratory at the beginning of the 21st century.^8^ Notably, after highly insightful seminal contributions by Wiesner and co‐workers^9^ on delphinine‐type alkaloids, synthetic studies toward diterpenoid alkaloids lay practically dormant for almost thirty years. Only in the last two decades have these natural products come back into focus as targets for chemical synthesis.^10^ The lack of progress toward the diterpenoid alkaloids can likely be attributed to the challenge posed by the structural complexity of their multiply bridged polycyclic skeletons, as illustrated by the hexacyclic hetidines and the heptacyclic hetisines (Figure 2).

Given the complex framework of the diterpenoid alkaloids, a first level of analysis in generating a plan for their syntheses is to focus on the molecular skeleton. Hence, a bondset might be generated without regard to the functional groups. Clearly, to disregard functional groups in determining the bondset, or to postpone it to a later phase of synthesis planning, is unusual. Throughout the 20th century, chemists have adhered to synthesis strategies that prioritize functional group considerations (functional group oriented synthesis).^11^ However, nature follows a different scenario in the biosynthesis of terpenes and hence terpene‐derived (terpenoid) alkaloids:^12^ The molecular framework of terpenoid molecules is assembled first from a linear precursor containing all the carbon atoms in a “cyclase” phase, followed by a second “oxidase” phase, in which the skeleton is decorated with functional groups through oxygenation. In terpenoid alkaloids, the incorporation of nitrogen atoms is believed to occur through a Mannich condensation or Prins cyclization (with participation of a hydride), either immediately prior to or during the oxidase phase.5b

Unsurprisingly, the way in which terpenoids are constructed in nature has been challenging to mimic in the laboratory setting.^13^ Even though mimicking the “cyclase” phase through a cationic polyene cascade is well‐established,^14^ accompanying rearrangement steps (e.g. methyl shifts, Wagner–Meerwein‐type rearrangements) can be low yielding in some cases. However, the advent of position‐selective C−H activation/functionalization reactions has paved the way for several impressive syntheses of terpenoids.^15^ Thus, although the relatively limited ability to mimic the oxidase phase prevents synthetic chemists from pursuing a truly biomimetic approach to terpenoid synthesis at present, we may still take our cue from nature and consider the skeleton of these topologically complex molecules, independent of functional groups, in the initial phase of designing a retrosynthesis.

## 2 Where Does One Begin To Identify a Bondset?

**Figure 3:**
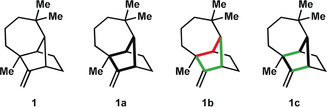
Bond‐network analysis of the maximally bridged ring of longifolene (**1**).

If retrosynthetic analyses were guided solely by the reactions one would seek to employ in the forward sense, they would be inherently biased toward transformations already known to the practitioner. On the other hand, topological analysis of the target, which is carried out without regard for functional groups, may identify different disconnections. Since the development of new modes of reactivity is a goal of synthesis, this more objective method can illuminate gaps in the known chemical space. In the case of bridged polycycles, such as longifolene (**1**; Figure 3), the most highly bridged ring incarnates the complexity of the system and is, thus, the focus of most topology‐based retrosyntheses of these scaffolds.

As the most highly bridged ring is necessarily central to a polycyclic ring system, it would seem reasonable to select this ring as the starting point for a synthesis, and to sequentially anellate all other rings to this central platform. Such an anellation approach would, however, lead to a synthesis with, at best, an inefficient linear increase in complexity.^16^

An alternative approach was pointed out by Corey and co‐workers in 1975.^18^ These authors recognized that retrosynthetic disconnection of certain bonds in the most highly bridged ring would lead to the largest reduction in structural complexity. To identify these bonds, one must first identify all the sites at which each primary ring^19^ is bridged, keeping in mind that an atom may be bridging relative to one ring but not to another. In the case of **1**, the maximally bridged ring is easily identified (see emphasis in **1 a**). For more highly bridged polycycles, it may be beneficial to automate this portion of the retrosynthesis, a feature that Corey and co‐workers built into LHASA^18^ and Sarpong and co‐workers have since adapted into a web‐based program.^20^

It is important to note that not every bond in the most highly bridged ring provides equal simplification upon disconnection. For example, disconnection of some bonds in **1** (marked red in **1 b**) would generate a precursor with a primary ring larger than seven‐membered, which is considered challenging to construct in the forward synthesis direction.^18^ Therefore, the remaining bonds (marked green in **1 b**) are designated as strategic. Further analysis^18^ must be applied to rank the merits and importance of the individual bonds marked in green. This additional analysis includes considerations of whether bridging is drastically reduced in the retrosynthetic direction, whether the change in complexity is maximized,^4^ and whether dangling substituents are minimized as one disconnects the strategic bonds.

For molecules that incorporate heteroatoms into their skeleton, separate consideration must be given to any C−X bonds to determine whether these bonds should be considered strategic.^18^ This is due to the relative ease of forming C−X bonds (the X group is inherently a functional group). Therefore, only a subset of Corey's rules apply. Importantly, C−X bonds may be considered strategic even if they are part of a large ring or one that is not maximally bridged.

The Corey network analysis essentially identifies which skeletal bond(s) of a target are best constructed last in a synthesis. It therefore defines the skeleton of a penultimate intermediate that lacks either the most highly bridged ring or a strategic C−X bond. This intermediate is subjected to network analysis to identify the strategic bond(s) for the next disconnection. In doing so recursively,^20^ one generates a complete bondset, as well as timing for each ring‐closing step in the forward synthesis. At one extreme, an open‐chain precursor containing all skeletal atoms of the target would be generated,^21^ and that precursor would then be advanced through *n* steps into a polycycle that possesses *n* rings, with each synthetic step closing one additional ring.

An even more rapid increase in complexity would be provided by reactions that form two skeletal bonds at a time, such as cycloadditions,^22^ or (if target‐relevant) greater than two bonds at a time. Such bicyclizations (or polycyclizations) close multiple rings in one stroke. When this concept is applied to the most highly bridged ring, bonds other than those well‐suited to one‐bond disconnection (cf. **1 b**) may be considered for two‐bond (bond pair) disconnection (see **1 c**). This version of Corey network analysis, which has been modified to allow consideration of multiple‐bond disconnections in the context of the maximally bridged ring,^23^ is referred to herein as bond‐network analysis.

Selecting one of several strategic bonds for disconnection is where we see that bond‐network analysis does not fully supplant the idea of functional group oriented synthesis, but instead complements it. Retrosynthetic disconnections should be chosen at this level to take advantage of inherent functionality in the target molecule where possible, thereby minimizing the number of peripheral modifications required in the forward synthesis. The introduction of additional functional handles (and the attendant increase in step count) should be balanced against their ability to facilitate the efficient formation of skeletal bonds.

This approach should enable a rational plan to synthesize complex polycyclic targets, such as the bridged, architecturally intricate diterpenoid alkaloids. A critical evaluation of eight relatively recent total syntheses of the hexacyclic hetidine and heptacyclic hetisine classes of alkaloids provides a sense of the state of the art. Particular attention is directed to how well each synthesis adheres to the concept of bond‐network analysis.

## 3 Hetidine Syntheses

**Figure 4:**
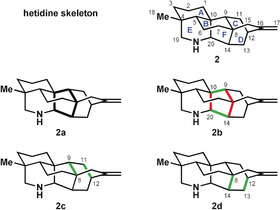
Bond‐network analysis of the maximally bridged ring of the hetidine skeleton. The primary rings of the hetidine scaffold have been defined as follows: A (C1‐C2‐C3‐C4‐C5‐C10); B (C5‐C6‐C7‐C8‐C9‐C10); C (C11‐C12‐C16‐C15‐C8‐C9); D (C12‐C13‐C14‐C8‐C15‐C16); E (C4‐C5‐C10‐C20‐N‐C19); F (C8‐C9‐C10‐C20‐C14).

In the hetidine skeleton (**2**, Figure 4), ring F is recognized as the most highly bridged ring (see emphasis in **2 a**). This ring is joined to an azabicyclo[3.3.1]nonane skeleton (rings A and E) as well as to a bicyclo[2.2.2]octane ring system (rings C and D). Hence, retrosynthetic disconnection of either the C9−C10 or C14−C20 bond in ring F would provide the greatest simplification (see **2 b**) if the analysis were restricted to one‐bond disconnections. The set of strategic bonds also includes the two C−N bonds.

A different picture arises when two‐bond disconnections (such as the Diels–Alder transform) are considered. To attain maximal retrosynthetic simplification, one of the rings targeted for the two‐bond disconnection should still be the most highly bridged ring. Of note, bonds targeted for two‐bond disconnection may differ from those designated as strategic for one‐bond disconnection. Examples of possible two‐bond disconnections of the hetidine skeleton are shown in **2 c** and **2 d**.

To address the question of where the community currently stands with regard to achieving the total synthesis of these bridged polycycles, we analyze the reported syntheses of hetidine derivatives. These analyses emphasize the framework‐forming transformations of intermediates that already contain most or all of the skeletal atoms.

**Scheme 1:**
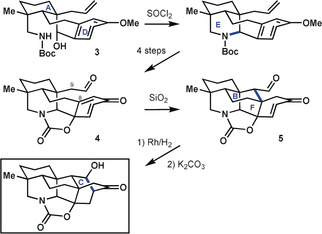
Key cyclization steps from the synthesis of a hetidine core structure by Sarpong and co‐workers.^24^

In their synthesis of a hetidine core structure, Sarpong and co‐workers^24^ sought to employ a late‐stage formal bicyclization to construct the [2.2.2] bicycle resident in the framework (Scheme 1). The key bond‐forming process started from precursor **3** that contained rings A and D, which were connected by parts of rings B and F. From there, they constructed the azabicyclo[3.3.1]nonane system by closing ring E. Thereafter, a series of refunctionalization steps led to dearomatized intermediate **4**. Isomerization to **5** closed rings F and B by forming the C8−C9 bond. Although not considered strategic for one‐bond disconnection in the full hetidine scaffold (as doing so would form a macrocycle), the C8−C9 bond is strategic in scaffolds where ring C is not yet closed, as is the case here. The latter ring was closed in the final ascent from **5** to give the heptacyclic target. This synthesis followed a retrosynthetic plan that was identified using bond‐network analysis, which suggested a late‐stage closure of rings C and F through a bicyclization transformation (cf. **2 c**). However, the analysis did not predicate a one‐step bicyclization, but rather left room for variants in the synthetic direction.

**Scheme 2:**
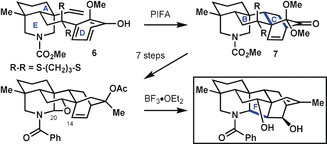
Late‐stage ring formation steps in the synthesis of a hetidine derivative by Qin and co‐workers.^25^ PIFA=phenyliodine(III) bis(trifluoroacetate).

In their approach to the hetidine framework, Qin and co‐workers^25^ started the late‐stage cyclization phase of their synthesis from a tricyclic precursor (**6**; Scheme 2) that already possessed an azabicyclo[3.3.1]nonane system (rings A and E) and an attached ring D. The first key transformation was a bicyclization that simultaneously formed rings B and C to give ketal **7**. Then followed seven refunctionalization steps before ring F was closed at the strategic C14−C20 bond. In accordance with bond‐network analysis, the final ring formed in the Qin approach was the maximally bridged ring. A well‐precedented oxidative dearomatization/intramolecular Diels–Alder cascade^26^ was applied to forge the bicyclo[2.2.2] framework that preceded the final bond‐forming step to construct the hetidine skeleton.

**Scheme 3:**
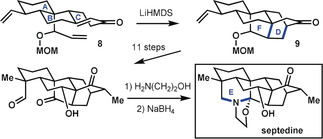
Synthesis of hetidine‐type natural product septedine by Li and co‐workers.^27^ LiHMDS=lithium hexamethyldisilazide, MOM=methoxymethyl.

The approach by the Li group to the highly bridged polycyclic framework of septedine^27^ started from tricyclic precursor **8**, in which rings A, B, and C were already anellated (Scheme 3). Bicyclization simultaneously closed rings D and F to give pentacycle **9**. This Diels–Alder‐type cycloaddition deviates from that seen in the other alkaloid syntheses discussed here in that it is initiated by formation of an enolate rather than by oxidative dearomatization to a masked *o*‐quinone. With **9** in hand, several steps were undertaken to adjust peripheral substituents, before ring E was closed in a final sequence to access septedine. The Li approach conformed to bond‐network analysis in that a bicyclization is employed to construct the maximally bridged ring in the penultimate cyclization step of the synthesis, after which ring E is closed by forging strategic C−N bonds. In addition, the synthesis by Li and co‐workers contained elements of a potentially biomimetic approach in that the nitrogen atom was incorporated at a late stage through a double reductive amination with ethanolamine.

**Scheme 4:**
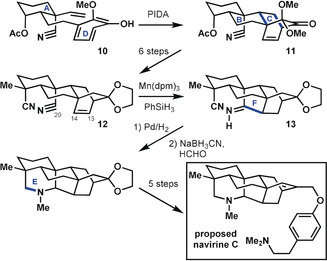
Synthesis of the proposed structure of navirine C by Liu and Ma.^28^ dpm=dipivaloylmethanato, PIDA=phenyliodine(III) diacetate.

Liu and Ma^28^ addressed the synthesis of the proposed structure of navirine C in a synthesis (Scheme 4) that conceptually resembles that of Qin and co‐workers,^25^ although these efforts were likely pursued contemporaneously. The conceptual similarities are readily apparent in both the retrosynthetic disconnections chosen and how starting material **10** for the synthesis by Liu and Ma resembles compound **6** in the synthesis by Qin and co‐workers. However, in the former case, ring E had yet to be closed, thereby avoiding complications that would have arisen regarding position‐selective (C19 versus C20) attachments to ring E.

The first step involved the well‐established oxidative dearomatization of ring D, which enabled formation of the [2.2.2] bicycle within **11** and the simultaneous closure of rings B and C. At this stage, following five refunctionalization steps to generate **12**, Liu and Ma employed a hydrogen‐atom transfer^29^ to the C13−C14 double bond to close ring F through the strategic C14−C20 bond. This process generated **13** with the necessary functionality for the concluding formation of ring E. Overall, this synthesis may be thought of as a hybrid approach that combines elements of bond‐network analysis with bio‐inspiration. The nitrogen atom resident in the hetidine framework is introduced early in the form of a nitrile group, which is critical to the later construction of the maximally bridged ring F in a manner somewhat reminiscent of the Mannich approach proposed for the biosynthesis of the related aconitine skeleton.^30^ In this way, the disconnections utilized by Liu and Ma are potentially biomimetic, but the actual reactions employed in the forward sense are quite dissimilar from the conditions likely in the biosynthesis.

**Figure 5:**
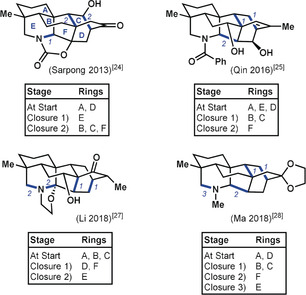
Comparison of ring‐closure strategies employed in the syntheses of hetidine scaffolds.

To facilitate comparison of the four syntheses of hetidine derivatives, the following overview illustrates the bondset and timing adopted by each group for the assembly of the hexacyclic skeleton (Figure 5):

A dominant feature in all four syntheses is the focus on a cycloaddition approach to the bicyclo[2.2.2]octane portion (rings C and D) of the hetidines. Each synthesis incorporates one of these two rings at an early stage to facilitate a bicyclization. Additionally, none of the syntheses started with a completed ring F, which was closed somewhere along the synthesis route. To what extent did the individual researchers avail themselves of bond‐network analysis? This would be reflected in the closure of ring F in the last skeletal C−C bond‐forming step. This holds for the synthesis by Qin and co‐workers^25^ of a hetidine derivative which featured early installation of the nitrogen‐containing ring E, then followed an optimal plan to assemble the hexacyclic skeleton, ending with formation of the strategic C14−C20 bond. Likewise, Liu and Ma^28^ formed the C14−C20 bond as part of the endgame in conjunction with the closure of ring E at the C19−N bond. These approaches are contrasted by the syntheses executed by the groups of Sarpong^24^ and Li,^27^ in which the researchers instead closed ring F through bicyclization strategies that utilized bonds that were not considered strategic for one‐bond disconnection, and would thus be excluded under Corey's original network analysis. Nonetheless, all four syntheses adhere to the suggestions of the expanded bond‐network analysis in their own way, thus showcasing how this type of analysis guides retrosyntheses without prescribing the transformations to be used in the forward synthesis.

## 4 Hetisine Syntheses

**Figure 6:**
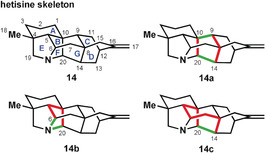
Bond‐network analysis of the maximally bridged ring of the hetisine skeleton. The primary rings of the hetisine scaffold have been defined as follows: A (C1‐C2‐C3‐C4‐C5‐C10); B (C5‐C6‐C7‐C8‐C9‐C10); C (C11‐C12‐C16‐C15‐C8‐C9); D (C12‐C13‐C14‐C8‐C15‐C16); E (C4‐C5‐C6‐N‐C19); F (C5‐C6‐N‐C20‐C10); G (C8‐C9‐C10‐C20‐C14).

The heptacyclic hetisine skeleton (**14**; Figure 6) contains two primary rings (F and G, labeled differently from hetidine) which are equally highly bridged. Application of bond‐network analysis^18^ to the hetisine skeleton unveils two strategic bonds (C14−C20 and C9−C10, see **14 a**) in ring G, as seen previously in the hetidine analysis (cf. **2 a**). Bond‐network analysis of the other maximally bridged ring (ring F) reveals two additional strategic bonds (N−C6 and N−C20), as indicated in **14 b**.

As examination of the two primary rings (i.e. F and G) did not point to a singularly attractive synthetic pathway, it would be appropriate to extend the analysis to a four‐ to seven‐membered secondary ring.^18^ For the hetisine skeleton (**14**), this applies to the highly bridged envelope of rings B and G (cf. **14 c**). However, bond‐network analysis doesn't reveal any further options for late‐stage closure of this ring; the only strategic bond is shared with ring G. Instead, since many bonds in this ring are red‐flagged for one‐bond disconnection—and here is a twist of the network analysis—this ring could instead be generated early and maintained throughout the synthesis as the majority of strategic disconnections would leave it intact.

The existing syntheses of hetisine natural products and their closely related derivatives are summarized in the following schemes, with emphasis on transformations that contribute directly to the formation of skeletal bonds.

**Scheme 5:**
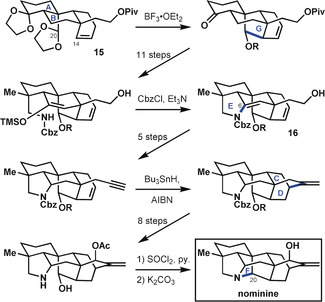
Synthesis of hetisine‐type diterpenoid alkaloid nominine by Muratake and Natsume.^31^ AIBN=azobisisobutyronitrile, Cbz=benzyloxycarbonyl, Piv=pivaloyl, py.=pyridine.

In their synthesis of the hetisine alkaloid nominine, Muratake and Natsume^31^ utilized tricyclic precursor **15** comprising rings A, B, and an envelope of rings C and D of the target structure (Scheme 5). They rigidified the system by closing the C14−C20 bond, namely ring G. A lengthy sequence following formation of the C14−C20 bond culminated in the closure of ring E by forming the C6−N bond to give carbamate **16**. Muratake and Natsume then addressed the assembly of the bicyclo[2.2.2] ring system (rings C and D), followed by several steps to adjust the functionality therein. It was only at the very end of the synthesis that the C20−N bond was formed to close ring F, in accord with bond‐network analysis (cf. **14 b**). Although the penultimate ring closure to form ring C did not involve the maximally bridged ring at that stage, all other ring‐closing steps did align with the bond‐network analysis.

**Scheme 6:**
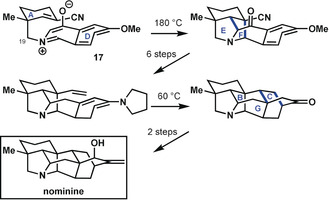
Late‐stage cyclization steps in the synthesis of nominine by Peese and Gin.^32^

A second synthesis of nominine by Peese and Gin^32^ followed a drastically different approach, which focused on early generation of the two most highly bridged rings (Scheme 6). The advanced intermediate (**17**) that was employed contained ring A connected through C19 to an isoquinolinium moiety, representing ring D and elements of rings F and G. The ascent to the nominine skeleton was initiated through an inventive 1,3‐dipolar cycloaddition that closed rings E and F. After six refunctionalization steps, a second bicyclization closed rings B, C, and G in a Diels–Alder reaction. In both bicyclization steps, bonds were formed that were undesirable for one‐bond disconnection approaches based on bond‐network analysis (see Figure 6). Foremost, this synthesis impresses by its rapid increase in target‐relevant complexity, advancing from the starting tricycle to the heptacyclic hetisine structure in only two skeleton‐building steps. This underscores the power of bicyclization reactions in the synthesis of bridged polycyclic targets.

**Scheme 7:**
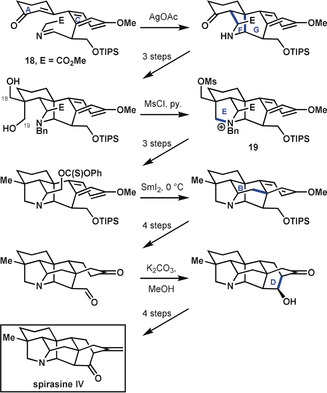
Introduction of bridged rings in spirasine IV by Zhang et al.^33^ Bn=benzyl, Ms=mesyl, TIPS=triisopropylsilyl.

In their synthesis of spirasine IV, Zhang et al.^33^ initiated their final set of ring‐forming reactions from the rather simple precursor **18** (Scheme 7), which possesses only two directly connected rings (A and C). Essential skeletal atoms for rings B, D, F, and G were attached to this bicycle, thereby facilitating their subsequent assembly. The ring formations were initiated by a 1,3‐dipolar cycloaddition to form rings F and G, a similar approach as in the synthesis by Peese and Gin.^32^ At this stage, the remaining atoms, C18 and C19, of ring E were introduced, and ring E was closed to afford pentacyclic intermediate **19**. After a few refunctionalization steps, ring B was closed through a free‐radical addition to the aromatic ring C. Another series of functional group adjustments set the stage for an aldol reaction to close ring D and completion of the synthesis in four additional steps. Overall, the synthetic approach adopted here is of the anellation type, where “construction of the most highly bridged core occurs first followed by subsequent crocheting of the remaining rings”. Nonetheless, the synthetic ascent to the hetisine skeleton as a target is marked by a steady increase in complexity over the four steps that lead to the skeleton. It eschews the principles of bond‐network analysis, but profits from a well‐chosen starting point.

**Scheme 8:**
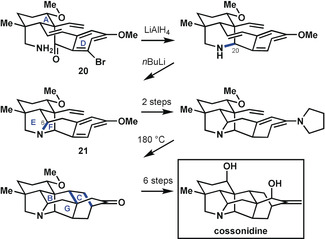
Closure of bridged rings in the synthesis of cossonidine by Sarpong and co‐workers.^34^

In their synthesis of cossonidine, Sarpong and co‐workers^34^ drew upon the group's prior synthesis^24^ of a navirine precursor by using a tricyclic starting compound (**20**; Scheme 8) containing rings A and D connected by an envelope of rings B and G, thereby realizing the analysis depicted by **14 c**. As in the previous synthesis of the hetidine framework, they first forged the C20−N bond. This was followed immediately by formation of the C6−N bond, which closed rings E and F to give pentacycle **21**. The ultimate heptacyclic skeleton was reached following the approach used by Peese and Gin^32^ to close rings B and C in a Diels–Alder reaction. Finally, the functionalities were adjusted in a series of redox manipulation steps to reach cossonidine in a sequence that does not quite give an exponential increase in complexity, but does fully align with bond‐network analysis.

**Figure 7:**
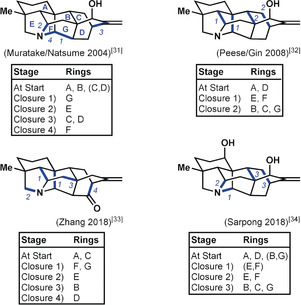
Comparison of ring‐closure strategies in the syntheses of hetisine‐type natural products. Herein, an envelope of two rings is denoted using the two ring letters in parentheses and separated by a comma. For example, (C,D) refers to an envelope of rings C and D.

To facilitate a comparison of the four syntheses of hetisine derivatives in the context of bond‐network analysis, the following overview illustrates the bondset and timing of bond formation chosen by the different groups for the assembly of the heptacyclic skeleton (Figure 7):

As with the hetidine syntheses, bicyclizations are embraced in constructing the hetisine skeleton. These powerful transformations dramatically reduce the number of ring‐closing steps required. Another unifying theme that emerged was the manner in which each research group leveraged the nitrogen atom inherent in the framework of the hetisines for strategic bond formation. This is immediately apparent in the three syntheses which utilize a C−N bond to close rings directly, but is also instrumental to the 1,3‐dipolar cycloaddition strategies used in the syntheses by both Zhang et al.^33^ and Peese and Gin.^32^

## 5 Summary and Outlook

Sections 3 and 4 detailed the state of the art in the synthesis of bridged polycyclic skeletons. To what extent did the scientists behind these syntheses avail themselves of the considerations outlined in Section 2? A full answer to that question would require knowledge of all disconnections considered, including the original retrosynthesis and any failed routes. Although this is unfortunately not feasible, one can still discern that the majority of the syntheses described here have several features in common, which provide insight into the elements of a successful synthesis.

First, each of the syntheses of bridged polycyclic diterpenoid alkaloids covered here comprise two phases. The first phase addresses reaching a fully assembled “base camp” (i.e. a key intermediate compound), which already contains a number of connected rings (in the present examples, two or three), but with the rigidifying bridges yet to be installed. Attached to the skeleton of the key intermediate are side chains, which provide the skeletal atoms of the yet‐to‐be‐closed rings. The second phase of the syntheses comprises the elevation of the key intermediate to the desired hexa‐ or heptacyclic skeleton of the target by forming strategic bonds to forge the bridging rings. Although “phase one” efforts are not reviewed in the present context, the efforts of “phase two” are highlighted, albeit without significant discussion relating to the functionality present in the target structures.

A second trend apparent across the eight syntheses is the preference for bicyclization reactions, which achieve particularly rapid increases in target‐relevant complexity. In seven of the eight examples, bicyclization reactions were employed, most often for the construction of the bicyclo[2.2.2]octane ring system present in both the hetidine and hetisine scaffolds.

The anellation approach to build bridged polycyclic compounds by the early introduction of the maximally bridged ring (as discussed in Section 2) is adumbrated in only one of the eight syntheses covered (spirasine IV by Zhang et al.^33^). The remainder of the syntheses that were surveyed largely follow bond‐network analysis at the retrosynthetic level, even if these disconnections did not translate perfectly to the forward synthesis (see the synthesis of the hetidine core by Sarpong and co‐workers^24^). It is our opinion that a bond‐network analysis approach to retrosynthesis is not consistently employed or considered by the synthetic community at present, but that the degree to which the precepts of bond‐network analysis are adhered in reported syntheses nevertheless validates the principles outlined in Section 2.

On the other hand, bond‐network analysis may not necessarily be the most effective approach in all cases, depending on the functional groupings on a target molecule. This situation is particularly appreciated in light of previous reports,^35^ in which solely following a bond‐network analysis in forward syntheses led to conflicts with the functionalities that needed to be generated. However, there is value in the fact that bond‐network analysis sometimes suggests disconnections where the forward synthesis has no precedent and a new reaction may need to be developed. Finding creative solutions to challenging problems that may arise in this manner is the lifeblood of organic synthesis. Reaction development enables synthesis, which in turn highlights areas where further reaction development is needed. That cycle advances the frontiers of chemistry and can be driven forward by looking for strategies to accomplish these objectively identified disconnections, especially when we challenge ourselves to exploit native functionality to do so.

On the basis of the widespread application—conscious or unconscious—of bond‐network analysis in successful syntheses of hetidine‐ and hetisine‐type diterpenoid alkaloids, one should consider undertaking a bond‐network analysis when planning the synthesis of a bridged polycyclic target structure, performing recursive network analyses to obtain hints on disconnections to give key intermediates. Searching within these for hints of the potential application of bicyclization strategies is of paramount importance in assuring a rapid increase in complexity. The final stage in the planning process would then be to ascertain the compatibility of the intended bondset with the functionalities present in the target structure, as well as to consider which transformation and functional group based strategies may be employed to capitalize upon the disconnections identified through bond‐network analysis.

## Conflict of interest

The authors declare no conflict of interest.

## Biographical Information

*Nicolle A. Doering received her B.S. in Chemistry from the University of California‐Santa Barbara in 2015. During that time she carried out research with Prof. Armen Zakarian on enantioselective α‐functionalization of carboxylic acid derivatives, as well as on enantioselective total synthesis. She is currently a Ph.D. student in the Chemistry program at the University of California‐Berkeley, under the supervision of Prof. Richmond Sarpong. Her current research is focused on strategies to enable the total synthesis of diterpenoid alkaloid and terpene natural products*.

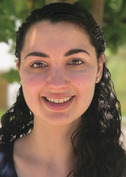

## Biographical Information

*Richmond Sarpong was born in Ghana, West Africa in 1974. He received his B.A. from Macalester College (St. Paul, USA) in 1995, where he carried out research with Prof. R. Hoye. He completed his Ph.D. in 2000 with Prof. M. F. Semmelhack at Princeton University (USA). After three and a half years as a postdoctoral fellow with Prof. B. Stoltz at Caltech (Pasadena, USA) he began his independent career at the University of California, Berkeley, where he is at present Professor of Chemistry. His research includes the development of new strategies for the synthesis of complex natural products including the diterpenoid alkaloids*.

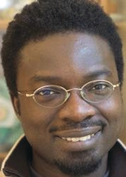

## Biographical Information

*Reinhard W. Hoffmann studied chemistry from 1951 to 1958 at the University of Bonn, where he completed his Ph.D. with Prof. B. Helferich. Two years of postdoctoral studies at the Pennsylvania State University were followed by a second position with Prof. G. Wittig at the University of Heidelberg, where he habilitated in 1964. In 1967 he was appointed as Dozent at the TH Darmstadt. Since 1970 he has been Professor of Organic Chemistry at the Universität Marburg (emeritus since 2001). He was a visiting professor at the Universities of Wisconsin, Bern, California at Berkeley and at Santa Barbara, as well as at Kyoto University*.